# ErbB Receptor Feedback Inhibitor 1 Mutation in Biliary Tract Cancers: Turning Resistance Into Response

**DOI:** 10.1200/PO-25-00673

**Published:** 2026-06-03

**Authors:** Supriya Peshin, Fen Saj, Pashtoon M. Kasi, Felicity David, Janio Szklaruk, Quentin Kimana, Shubham Pant, Sunyoung S. Lee, Jennifer J. Knox, Mitesh J. Borad, Milind Javle

**Affiliations:** ^1^ Department of Internal Medicine, Norton Community Hospital, Ballad Health, Norton, VA; ^2^ Department of Gastrointestinal Medical Oncology, The University of Texas MD Anderson Cancer Center, Houston, TX; ^3^ Department of Oncology and Therapeutics Research, City of Hope Orange County, Irvine, CA; ^4^ Department of Diagnostic Radiology, The University of Texas MD Anderson Cancer Center, Houston, TX; ^5^ Department of Medical Oncology and Hematology, Princess Margaret Cancer Center, Toronto, ON, Canada; ^6^ Division of Hematology & Oncology, Department of Medicine, Mayo Clinic, Scottsdale, AZ

## Abstract

**PURPOSE:**

*EGFR* alterations occur in a subset of biliary tract cancers (BTCs) and are often linked to disease progression and poor prognosis, yet targeted approaches remain underexplored. *ERRFI1*, encoding the epidermal growth factor receptor (EGFR) inhibitor MIG6, is mutated in various cancers, including BTC, representing a potential predictive biomarker. We investigated whether loss of *ERRFI1* function may enhance sensitivity to EGFR-targeted tyrosine kinase inhibitors (TKIs), presenting a potential therapeutic opportunity.

**METHODS:**

This is a retrospective multicenter study of patients with BTC harboring *ERRFI1* alterations. Data were extracted from electronic health records following institutional approval. *ERRFI1* alterations were classified based on the alteration type, variant allele frequencies were documented, and coalterations were analyzed. Treatment outcomes including response, time to progression (TTP), overall survival (OS), safety profiles, and tumor marker kinetics were noted.

**RESULTS:**

Fourteen BTC patients with *ERRFI1* mutations were identified in our database; the median age was 50 years, 64% were males; all cases were intrahepatic cholangiocarcinomas. Common coalterations included *IDH1* (36%), *TP53* (29%), and *ARID1A* (29%). All patients received gemcitabine-cisplatin as first-line treatment, and 79% received immune checkpoint inhibitors. Eight patients (57%) received EGFR TKIs. Best responses to EGFR therapy included three partial responses (2 responses lasting >20 months), four stable diseases, and one progressive disease. The median TTP was 7 months (95% CI, 6 to 21 months). Treatment was well-tolerated: toxicities included rash (n = 2), transaminitis (n = 1), and diarrhea (n = 1). The median OS was 20 months (95% CI: 8-36 months); 50% remained alive at last follow-up.

**CONCLUSION:**

*ERRFI1* mutations represent actionable biomarkers in BTC, with EGFR-targeted therapy demonstrating meaningful clinical benefit in this heavily pretreated population. These findings support integration of *ERRFI1* testing into routine molecular profiling of BTC and other EGFR-driven malignancies.

## INTRODUCTION

Biliary tract cancers (BTCs), some of the most challenging malignancies in oncology, are characterized by their aggressive behavior and limited treatment options.^1^ While first-line chemoimmunotherapy offers modest survival benefits, most patients progress, underscoring the need for effective second-line strategies.^2^ Recent advances in comprehensive genomic profiling have revealed actionable molecular targets in nearly 40% of cases. However, many patients lack these targets or develop resistance, highlighting the need for additional biomarkers and therapeutic avenues.^3,4^

**CONTEXT**

**Key Objective**
Can loss-of-function mutations in *ERRFI1* predict response to EGFR-targeted therapy in biliary tract cancers (BTCs)?
**Knowledge Generated**
In this multicenter retrospective study of 14 patients with *ERRFI1*-mutant intrahepatic cholangiocarcinoma, EGFR tyrosine kinase inhibitor therapy demonstrated meaningful clinical activity in heavily pretreated patients. Among eight patients receiving EGFR-directed therapy, seven achieved disease control (three partial responses, four stable disease) with a median time to progression of 7 months. Treatment was well-tolerated with manageable toxicities.
**Relevance**
*ERRFI1* mutations define a rare but actionable molecular subset of biliary tract cancers responsive to EGFR inhibition. These findings support integration of *ERRFI1* testing into routine next-generation sequencing panels for BTCs and potential tumor-agnostic treatment strategies across *ERRFI1*-mutant malignancies.

Epidermal growth factor receptor (EGFR) pathway dysregulation contributes significantly to BTC pathogenesis.^5^ EGFR-targeted approaches have not been successful for treatment in unselected BTC populations.^6,7^ *EGFR* mutations occur infrequently, but have been reported in gallbladder cancer, where they represent actionable targets.^8,9^ An under-recognized but potentially important component of this signaling axis is the ErbB receptor feedback inhibitor 1 (*ERRFI1*) gene, which encodes mitogen-inducible gene 6 (MIG6), a cytoplasmic adaptor protein that serves as a key negative regulator of EGFR activity. In addition to EGFR, *ERRFI1* also downregulates other ErbB family members and modulates MET receptor signaling. Emerging data further implicate *ERRFI1* in broader cellular roles, including stress response, cell cycle regulation, and possible nuclear functions, highlighting a more expansive functional profile than previously appreciated.^10-13^

Although *ERRFI1* alterations are uncommon in patients with BTC, emerging preclinical data and anecdotal case reports suggest that this could represent an actionable biomarker.^14^ An estimated 1% of BTCs harbor this mutation (source: AACR Genie).^15,16^ Loss of *ERRFI1* function through somatic mutations, deletions, or epigenetic silencing disrupts feedback inhibition, unleashing sustained EGFR signaling in cholangiocytes.^17,18^ This biological vulnerability may be therapeutically targeted by EGFR tyrosine kinase inhibitors (TKIs). Preclinical and anecdotal evidence suggests that tumors with *ERRFI1* loss may exhibit increased sensitivity to EGFR-targeted therapies like erlotinib.^9,19^ However, systematic clinical validation in larger cohorts remains limited, representing a knowledge gap in precision oncology for BTCs.

Given the molecular heterogeneity of BTCs and the limited efficacy of current therapies, the identification of novel molecular subsets remains a clinical priority. In this study, we describe the clinical, molecular, and therapeutic features of patients with *ERRFI1*-mutant BTC, including patient studies of responses to EGFR-targeted therapy, to highlight the potential of *ERRFI1* as a predictive biomarker for treatment selection.

## METHODS

This retrospective case series includes patients with histologically confirmed BTC harboring *ERRFI1* alterations treated at the MD Anderson Cancer Center, City of Hope, and Mayo Clinic between January 2015 and February 2025. Data were extracted from electronic health records after obtaining institutional approval and/or informed consent from patients at all three centers. Data collected included patient demographics, clinical and tumor characteristics, genomic alterations, therapeutic interventions, and clinical outcomes. Genomic data collected included *ERRFI1* alteration type (classified as loss-of-function [LOF, eg, frameshift, stop-gain, deletions, duplications], missense, or unknown significance), with variant allele frequencies (VAFs), nucleotide and amino acid changes, and coalterations prioritized for oncogenic drivers such as *IDH1, FGFR2,* and *TP53.* Microsatellite instability (MSI) status and tumor mutation burden (TMB) were recorded. Treatment data including surgical interventions, radiotherapy, and systemic therapies (chemotherapy, immunotherapy, and targeted agents) were included. Outcomes analyzed included responses, time to progression (TTP), overall survival (OS), toxicity of EGFR-TKIs, and tumor marker kinetics including CA 19-9 and circulating tumor DNA (ctDNA) dynamics where available. Statistical analysis used descriptive statistics to summarize patient and tumor characteristics, using Kaplan-Meier methods to estimate PFS and OS, censoring patients lost to follow-up or alive at last contact. To provide broader context for *ERRFI1* alteration frequency in BTCs and other cancers, we queried publicly available genomic databases including cBioPortal for Cancer Genomics^20^ and AACR Project GENIE.^21^

## RESULTS

A total of 14 patients with *ERRFI1*-mutated BTC were included in the study. The median age at diagnosis was 50 years (range, 24-71), nine (64%) were male, and most were white (92%). Common comorbidities included hyperlipidemia (42%), hypertension (33%), diabetes (33%), and metabolic-associated steatotic liver disease (MASLD, 33%). All patients had tumors of adenocarcinoma histology, with intrahepatic cholangiocarcinoma as the primary site. The mean CA 19-9 at diagnosis was 332.5 (4.9-1,998.6) IU/ml. Eight patients (57%) presented with localized disease, whereas six (43%) presented with metastatic disease at diagnosis. The prevalence of *ERRFI1* mutations in the MD Anderson Cancer Center institutional database was 0.59% (12 cases among 2,049 patients with BTC). The demographics and treatment characteristics are summarized in Table 1.

**TABLE 1.** Disease and Treatment Characteristics (N = 14)

Patient	Age/Sex	Stage at Diagnosis	Surgery (Y/N)	Radiotherapy (Y/N)	Systemic Therapy Lines	EGFR TKI (duration)	Best Response to EGFR TKI	Status (dead/alive)	OS (months)
1	71/M	IIIB	N	N	1. Gem-Cis-Durva 2. FOLFOX	NA	NA	Alive	19
2	71/M	IV	N	N	1. Gem-Cis 2. Erlotinib 3. FOLFIRI + afatinib 4. 5-FU + afatinib	Erlotinib (12 months) Afatinib (17 months)	SD	Dead	35
3	61/M	IV	N	Y	1. Gem-Ox-Durva 2. Erlotinib	Erlotinib (1 month)	PD	Dead	3
4	62/M	II^a^	Y	Y	1. Gem-Cis-Durva 2. Ivosidenib	NA	NA	Alive	17
5	29/M	IIIB	N	N	1. Gem-Cis-Durva	NA	NA	Alive	7
6	50/M	IV	N	N	1. Gem-Cis-abraxane 2. Nivo-Ipi	NA	NA	Dead	17
7	45/M	IV	Y	Y	1. Gem-Cis-Durva	NA	NA	Alive	20
8	24/F	IB^a^	Y	Y	1. Gem-Cis 2. Gem-Durva 3. Pemigatinib	NA	NA	Lost to follow-up	37
9	61/M	II^a^	Y	Y	1. Gem-Cis-Durva 2. Gem-Ox-Durva 3. Ivosidenib 4. Naliri-5FU 5. Erlotinib	Erlotinib (6 months—active)	SD	Alive	33
10	43/F	II^a^	Y	N	1. Gem-Cis-Durva 2. Erlotinib 3. FOLFOX	Erlotinib (21 months)	PR	Alive	42
11	58/F	IV	Y	Y	1. Gem-Cis-Durva 2. Ivosidenib 3. 5-FU + NALIRI + erlotinib	Erlotinib (6 months—active)	SD	Alive	23
12	51 years/F	II^a^	Y	Y	1. Gem-Cis 2. Erlotinib 3. Afatinib	Erlotinib (9 months) Afatinib (2 months)	SD	Dead	42
13	43 years/F	III	Y	Y	1. Gem-Cis 2. FOLFIRI 3. FOLFOX 4. Nivolumab off-label 5. Capecitabine 6. Gemcitabine 7. Erlotinib	Erlotinib (7 months)	PR	Dead	60
14	50 years/M	IV	Y	N	1. Gemcitabine-cisplatin 2. Pegylated hyaluronidase (PEGPH20) 3. Erlotinib	Erlotinib (4 months)	PR	Dead	16

Abbreviations: Durva, durvalumab; FOLFIRI, 5-fluorouracil, leucovorin, and irinotecan; FOLFOX, 5-fluorouracil, leucovorin, and oxaliplatin; Gem-Cis, gemcitabine and cisplatin; Gem-Ox, gemcitabine and oxaliplatin; NA, not applicable; NALIRI, nanoliposomal irinotecan; Nivo-Ipi, nivolumab and ipilimumab; OS, overall survival; PD, progressive disease; PR, partial response; SD, stable disease; TKIs, tyrosine kinase inhibitors.

^a^ Patients with localized disease were treated with systemic therapy at the time of disease progression.

**FIG 1.**
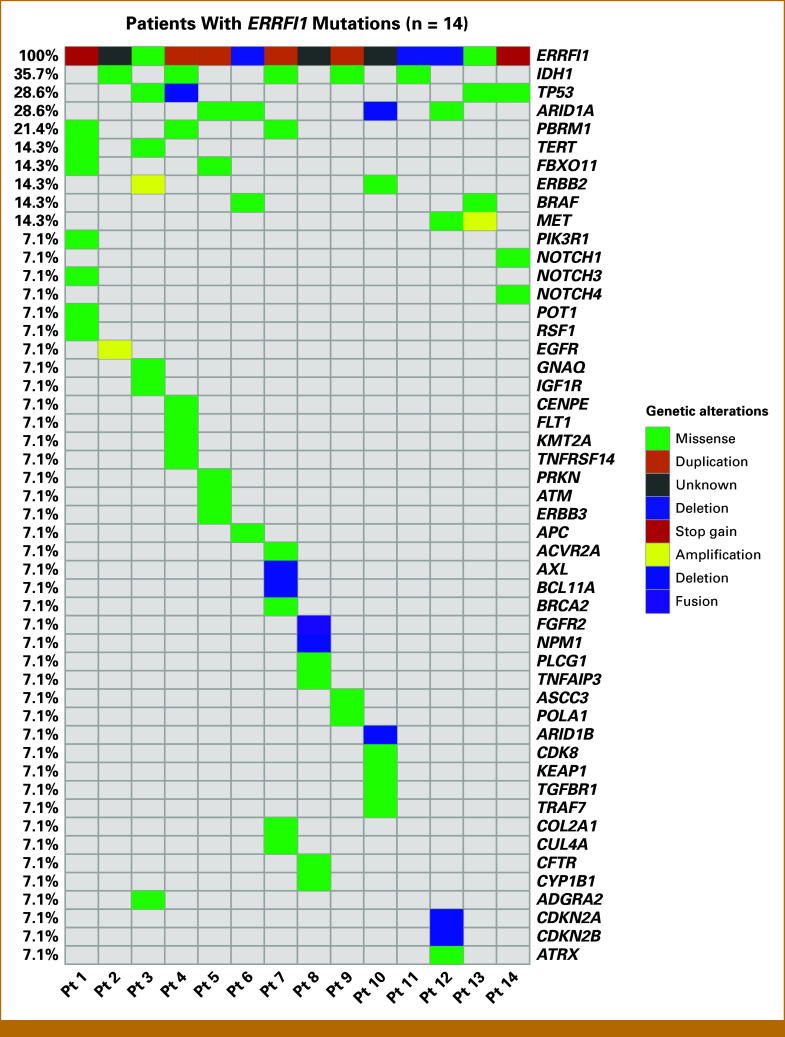
Genetic alteration landscape in *ERRFI1*-mutant BTCs. Oncoplot showing mutation profiles across 14 patients (Pt 1-Pt 14). Colors indicate alteration types per legend. Percentages show gene alteration frequency within the cohort. BTCs, biliary tract cancers.

All patients harbored *ERRFI1* alterations. Of these, 10 (72%) exhibited LOF variants, and two (14%) had missense mutations, whereas two (14%) cases had insufficient data to classify the variant type. Frequently observed coalterations included *IDH1* (36%), *TP53* (29%), *ARID1A* (29%), and *PBRM1* (21%) mutations. VAFs ranged from 0.12% to 39%. These mutational data are depicted in Figure 1 and Appendix Table A1.

**FIG 2.**
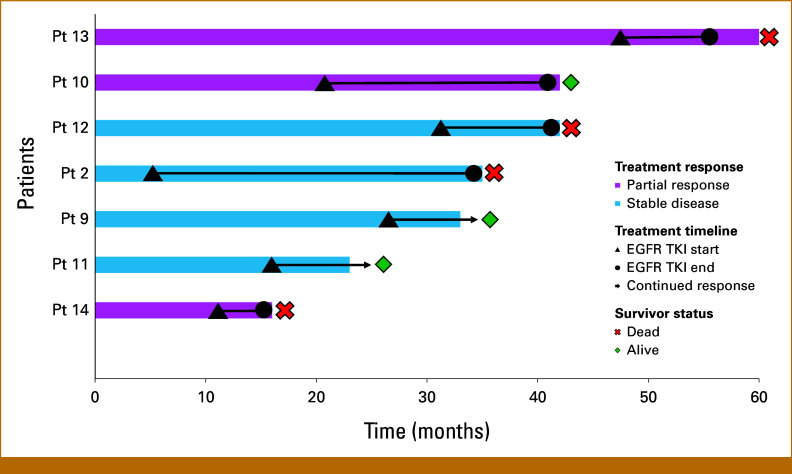
Treatment timeline and responses to EGFR-targeted therapy in *ERRFI1*-mutant patients. Swimmer plot depicting individual patient treatment courses with EGFR tyrosine kinase inhibitors. Treatment responses, timeline markers, and survival status are indicated per legend. EGFR, epidermal growth factor receptor; TKIs, tyrosine kinase inhibitors.

Nine patients (64%) underwent surgery, and eight (57%) received radiotherapy. All patients received gemcitabine and cisplatin as first-line therapy for metastatic disease. Eleven (79%) patients received immune checkpoint inhibitors, three received ivosidenib for *IDH1* mutations, and one received pemigatinib for an *FGFR2* fusion. Eight (57%) patients received EGFR TKIs. The decision for targeted therapy was based on multidisciplinary discussion and review of the literature. All eight received erlotinib 150 mg once daily, and two also received afatinib 40 mg once daily. Therapy was well-tolerated, with documented side effects being rash (n = 2), liver enzyme elevation (n = 1), and diarrhea (n = 1). Only one patient had intolerable toxicity (diarrhea) needing discontinuation. Treatment responses included partial response in three patients, stable disease in four, and progressive disease in one patient. Seven patients (50%) were alive at last follow-up (Table 1). For the overall cohort of *ERRFI1*-mutated BTC, the median OS was 20 (95% CI, 8 to 36) months. The median OS for patients receiving erlotinib was 34 months (95% CI, 17 to 47), whereas it was 18 months (95% CI, 9 to 30) for those not receiving erlotinib. Notably, among the seven patients who experienced disease stability or response from EGFR TKI therapy, the median TTP was 7 months (95% CI, 6 to 21 months; Fig 2).

**FIG 3.**
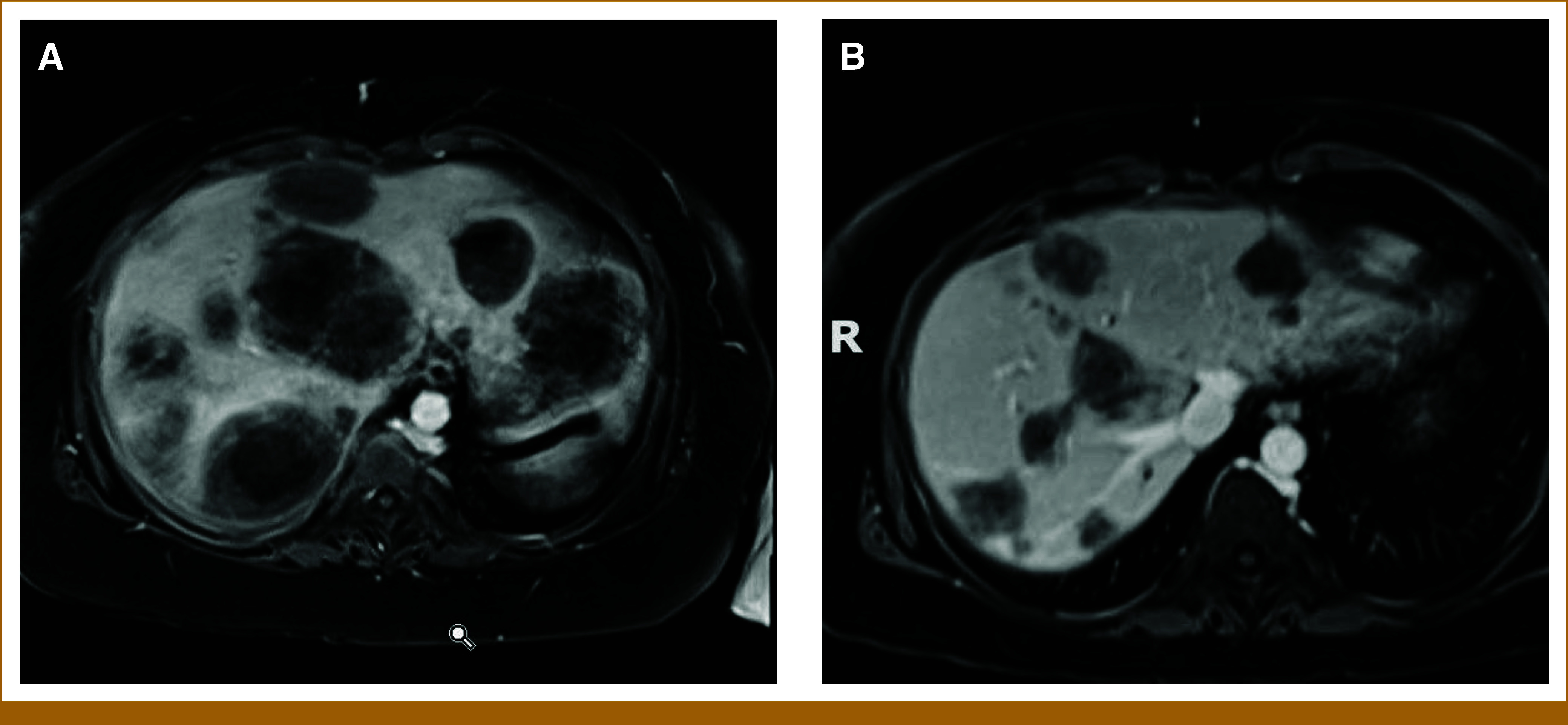
CT imaging showing treatment response to erlotinib in patient 13. Axial CT images (A) at baseline and (B) 2 months post erlotinib in an *ERRFI1*-mutant patient, demonstrating significant reduction in hepatic metastases. This represents a partial response in a heavily pretreated patient with refractory biliary tract cancer.

**FIG A1.**
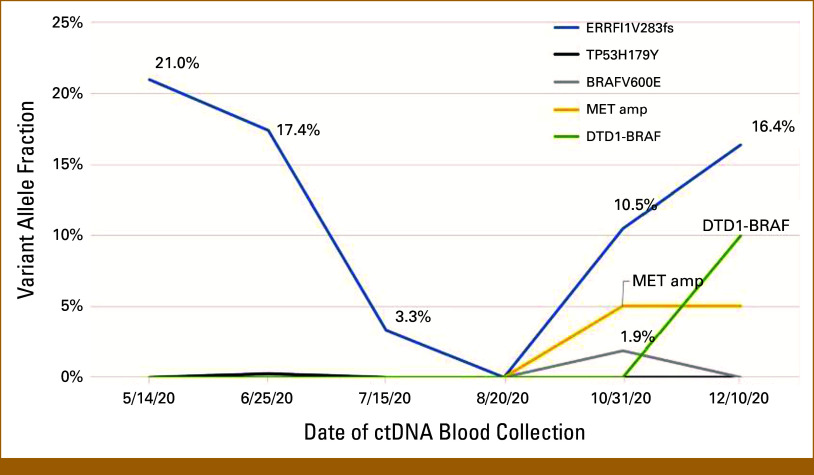
ctDNA kinetics during erlotinib therapy in patient 13. Serial ctDNA analysis showing *ERRFI1* V283fs variant allele fraction decline from 21.0% to 3.3% with erlotinib response, followed by rebound to 16.4% at progression. Acquired resistance mechanisms include emergent *MET* amplification and *DTD1-BRAF* fusion. Baseline *TP53* and *BRAF* mutations remained stable throughout treatment. ctDNA, circulating tumor DNA.

**FIG A2.**
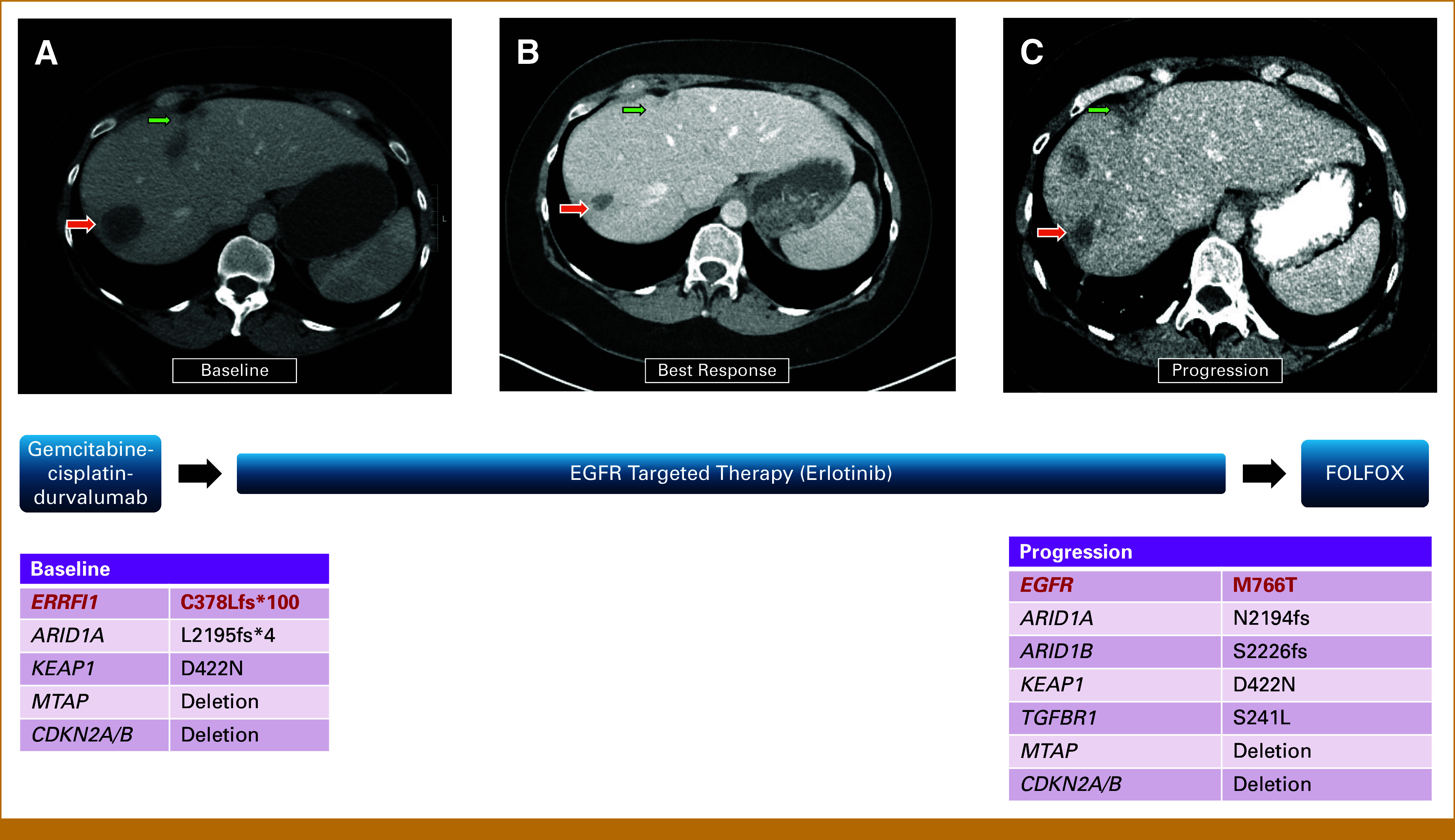
CT imaging and genomic analysis of patient 10 with metastatic intrahepatic cholangiocarcinoma. (A) Baseline CT showing (orange and green arrows) hepatic metastases; (B) best response to erlotinib therapy; and (C) disease progression. Tables display baseline and progression genomic profiling, highlighting baseline *ERRFI1* mutation and acquired EGFR M766I mutation at progression. EGFR, epidermal growth factor receptor.

A few cases are worth highlighting because of their exceptional responses to EGFR-targeted therapy in this cohort of heavily pretreated patients with BTC:

1. Patient 13 had chemorefractory disease after seven prior treatment lines and was previously advised to pursue supportive care with hospice. Despite this prognosis, the patient achieved a partial response lasting 7 months. Radiographic imaging demonstrated significant tumor shrinkage in multiple hepatic metastases (Fig 3), whereas ctDNA analysis revealed a parallel decline in *ERRFI1* variant allele frequency from 21.0% to 3.3% (Appendix Fig A1), correlating with clinical and radiographic improvement in performance status, nutritional parameters, and quality of life. On disease progression, liquid biopsy identified acquired resistance mechanisms including *MET* amplification and *DTD1-BRAF* fusion.
2. Patient 10 achieved a 21-month partial response to erlotinib with excellent tolerability. This patient harbored an *ERRFI1* C378Lfs*100 frameshift mutation and demonstrated substantial tumor regression across multiple hepatic metastases, as shown in the serial CT imaging (Appendix Fig A2). Notably, genomic analysis at disease progression revealed acquisition of an *EGFR* M766I resistance mutation, highlighting the evolution of resistance mechanisms during EGFR-targeted therapy.
3. Patient 2 maintained stable disease for 29 months across sequential EGFR inhibitors (erlotinib followed by afatinib) with an OS of 35 months.

These cases illustrate that even in the setting of advanced, refractory disease, *ERRFI1*-directed therapy may provide meaningful clinical benefit with sustained tumor responses and improved quality of life.

## DISCUSSION

Biliary tract cancer represents a poster child for precision medicine–based therapies.^22^ Genomic profiling has revealed that a substantial number of BTC cases harbor actionable alterations with potential therapeutic options, including (US) Food and Drug Administration–approved drugs, clinical trials, and guideline-endorsed off-label use.^23^ In this case series, heavily pretreated patients with somatic *ERRFI1* LOF mutations experienced sustained disease control with EGFR-targeted therapy, thereby providing clinical validation of the hypothesis that *ERRFI1*-deficient BTC relies on EGFR signaling for growth.^9,18^

**FIG 4.**
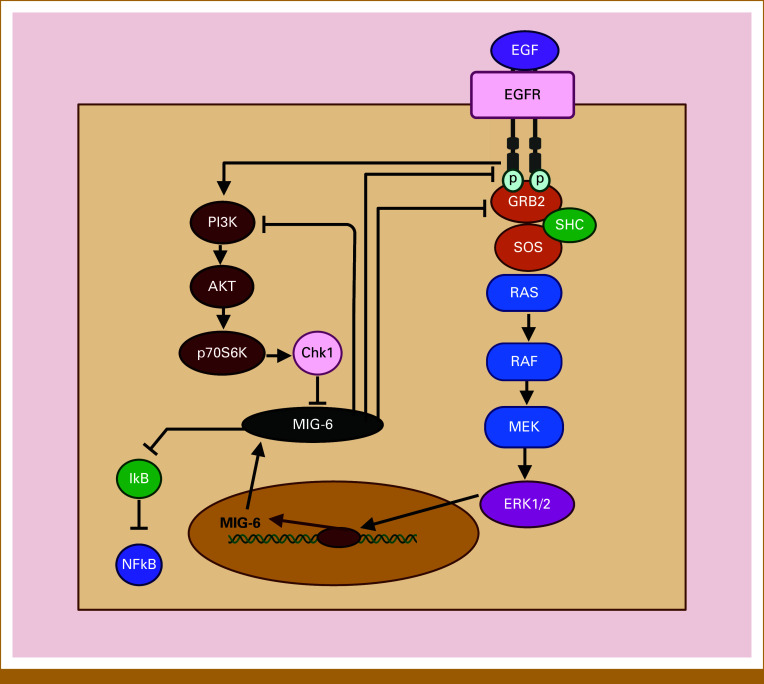
ERRFI1/MIG6 feedback inhibition of EGFR signaling. EGFR activation triggers downstream PI3K/AKT and MAPK pathways. ERK1/2 induces MIG6 transcription, which forms a negative feedback loop by directly inhibiting EGFR. Loss of ERRFI1/MIG6 disrupts this regulation, leading to sustained EGFR signaling and creating therapeutic vulnerability to EGFR inhibitors. AKT, protein kinase B; EGFR, epidermal growth factor receptor; MAPK, mitogen-activated protein kinase; MEK, mitogen-activated protein kinase kinase; RAF, rapidly accelerated fibrosarcoma; RAS, rat sarcoma virus; SOS, son of sevenless.

Located on chromosome 1p36.23, *ERRFI1* encodes MIG6, a cytoplasmic adaptor protein that fulfills a critical regulatory function by directly interacting with the intracellular domain of EGFR.^24,25^ Through this interaction, *ERRFI1* suppresses EGFR kinase activity, facilitates its internalization, and promotes degradation, thereby downregulating essential signaling cascades such as mitogen-activated protein kinase and phosphatidylinositol 3-kinase/protein kinase B (PI3K/AKT). This negative feedback loop is crucial for maintaining normal epithelial cell behavior and preventing uncontrolled cellular proliferation (Fig 4).^26,27^

Our cohort demonstrates the diverse spectrum of *ERRFI1* inactivating mechanisms. The predominant pattern involves frameshift mutations that create premature stop codons, eliminating crucial protein domains including the C-terminal ErbB-binding region.^10,26^ The G230 nonsense mutation similarly truncates the protein upstream of critical regulatory domains. These LOF alterations abolish EGFR regulatory capacity,^24^ whereas the A353V missense variant may disrupt protein-protein interactions within the ErbB-binding domain.^28^ The diversity of mutation types among responders suggests that any alteration disrupting MIG6 function may confer EGFR pathway dependence and sensitivity to EGFR TKIs. Mechanistically, *ERRFI1* loss creates context-dependent vulnerabilities. In EGFR-high tumors, loss enhances the pathway dependence and TKI sensitivity. In EGFR-low contexts, *ERRFI1* absence paradoxically activates AKT signaling through PH domain and Leucine-Rich Repeat Protein (PHLPP) phosphatase inhibition, suggesting that EGFR expression levels and *ERRFI1* status may serve as companion biomarkers for patient selection.^12,29,30^ Beyond direct EGFR effects, *ERRFI1* loss influences tumor immune microenvironments by promoting PD-L1 expression, while creating immunosuppressive conditions.^31,32^ This dual impact suggests that *ERRFI1*-deficient tumors may benefit from EGFR inhibitor-immunotherapy combinations, where pathway inhibition reverses immune suppression, whereas checkpoint blockade enhances T-cell activation. In addition, therapeutic options extend beyond erlotinib. Drugs like osimertinib, with enhanced central nervous system penetration and an improved toxicity profile,^33^ are compelling options for advanced BTCs, whereas combination approaches with AKT inhibitors or immunotherapy warrant systematic investigation. This is particularly relevant given the growing evidence that resistance mechanisms such as upregulation of insulin-like growth factor (IGF)-insulin receptor (IR; IGF2/IR/IGF1R) signaling and fibroblast-driven paracrine interactions may limit the durability of EGFR inhibition.^34,35^ These findings support the need for rational combination strategies to sustain responses.

The value of ctDNA and next-generation sequencing (DNA and RNA) for all patients with cholangiocarcinoma cannot be overemphasized.^36,37^ In addition, similar to any targeted therapies, rebiopsy or use of ctDNA/liquid biopsies to pick up additional actionable mechanisms of resistance and to monitor response can be helpful.^38^ In one of our patients, acquired resistance was related to *MET* aberration, which is a known actionable mechanism of resistance to EGFR-directed therapies.^39^

**FIG 5.**
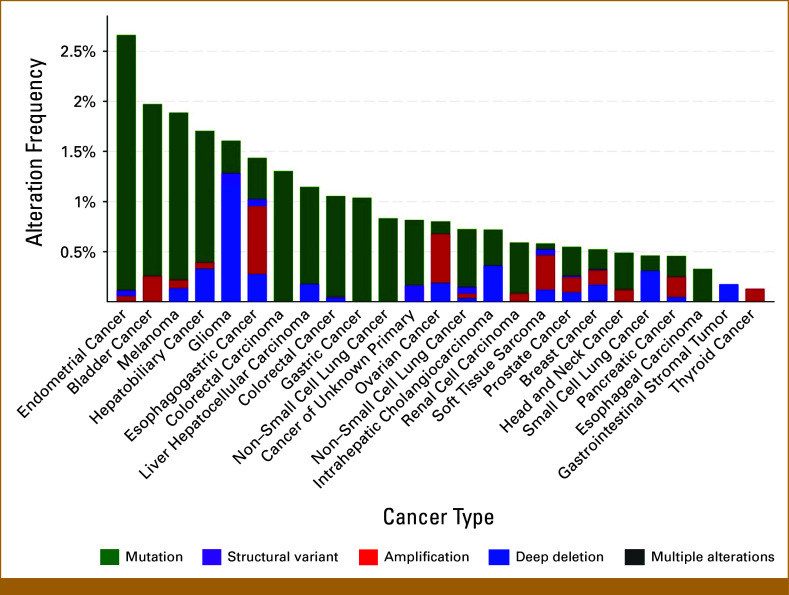
Pan-cancer frequency of *ERRFI1* alterations. *ERRFI1* alteration frequencies across cancer types, with alteration types indicated per legend.

*ERRFI1* alterations have been identified in multiple tumor types, including glioblastoma, triple-negative breast cancer, lung adenocarcinoma, and colorectal cancer, potentially rendering these malignancies susceptible to EGFR-targeted therapies (Fig 5).^25,29,40-43^ Furthermore, MIG6 loss has been associated with resistance to ALK and ROS1 TKI in non–small cell lung cancer.^44^ *ERRFI1* mutation has also been associated with afatinib resistance after prior stability with cetuximab in colorectal cancer.^43^ While *ERRFI1* alterations remain rare, the magnitude of clinical benefit observed may justify integration into routine molecular profiling.

This study has notable limitations, including small sample size, retrospective design, and heterogeneous patient population and treatment regimens. However, we believe that these limitations are inherent when investigating an uncommon genetic alteration of a rare cancer type, and our study population only included patients with intrahepatic cholangiocarcinoma. While prospective trials in such rare subsets are challenging, pooling and real-world evidence can play a critical role. This mirrors other successful precision oncology efforts, such as the aggregation of responses in *POLE*-mutant colorectal cancer, leading to practice guideline inclusion of immunotherapy, and the demonstration of benefit from EGFR-directed therapies in EGFR-amplified gastroesophageal cancers.^45,46^ These examples reinforce that even in rare subsets of uncommon diseases, collective clinical experiences can guide treatment, shape clinical trial design, and support broader tumor-agnostic strategies across oncology.

Conducting clinical trials within rare subsets of uncommon cancers represents a challenge. We believe that multi-institutional collaboration, molecular tumor boards, and basket trials may provide the necessary tools to investigate novel approaches in this setting. One of the key limitations in delivering targeted therapies is access to novel agents. In this study, the availability of affordable generics, such as those offered through Cost Plus pharmacy, was essential for enabling treatment in several patients.^47^

In summary, *ERRFI1* mutations define a rare but actionable subset of BTCs with meaningful responses to EGFR-targeted therapy. These findings support EGFR inhibition and clinical trial enrollment for these patients, with broader implications for tumor-agnostic treatment strategies.

## Data Availability

A data sharing statement provided by the authors is available with this article at DOI https://doi.org/10.1200/PO-25-00673.

## APPENDIX

**TABLE A1.** *ERRFI1* Mutation Profile

Patient	Mutation Type	Variant Allele Frequency	Genomic Location	Nucleotide Change	Amino Acid Change	MSI Status	TMB
1	Stop gain-loss of function	7.40%	NA	c.688G>T	p.G230*	MSS	3.7
2	Unknown	NA	NA	NA	NA	NA	NA
3	Missense	39%	Exon 4	c.1058C>T	p.A353V	MSS	5
4	Duplication-frameshift	19%	Exon 4	c.1134dupC	p.I379fs*6	MSS	4
5	Duplication-frameshift	7%	NA	c.723_751dup	p.S251fs	MSS	2.1
6	Deletion-frameshift	20%	Exon 4	c.353_356del	p.L118fs*2	MSS	3
7	Duplication-frameshift	22%	Exon 4	c.855dupC	p.R286fs*7	MSS	13
8	Unknown	NA	NA	NA	NA	MSS	4.2
9	Duplication-frameshift	16%	Exon 4	c.731dupG	p.R245fs*14	MSS	3
10	Duplication-frameshift	11.3%	Exon 4	c.1056_1132dup	p.C378Lfs*100	MSS	3
11	Deletion-frameshift	0.12%	NA	NA	p281fs*2	MSS	4
12	Deletion-frameshift	14%	NA	c.286del	p.L96fs*9	NA	NA
13	Missense	21%	NA	NA	p.V283fs	NA	NA
14	Stop gain-loss of function	11%	NA	NA	p.E384*	NA	NA

Abbreviations: MSI, microsatellite instability; MSS, microsatellite stable; NA, not applicable; TMB, tumor mutation burden.
